# Long-term treatment with metformin in obese, insulin-resistant adolescents: results of a randomized double-blinded placebo-controlled trial

**DOI:** 10.1038/nutd.2016.37

**Published:** 2016-08-29

**Authors:** M P van der Aa, M A J Elst, E M W van de Garde, E G A H van Mil, C A J Knibbe, M M J van der Vorst

**Affiliations:** 1 Department of Pediatrics, St Antonius Hospital, Nieuwegein, The Netherlands; 2 Department of Clinical Pharmacy, St Antonius Hospital, Nieuwegein, The Netherlands; 3 Department of Pediatrics, Jeroen Bosch Hospital, ‘s-Hertogenbosch, The Netherlands

## Abstract

**Background:**

As adolescents with obesity and insulin resistance may be refractory to lifestyle intervention therapy alone, additional off-label metformin therapy is often used. In this study, the long-term efficacy and safety of metformin versus placebo in adolescents with obesity and insulin resistance is studied.

**Methods:**

In a randomized placebo-controlled double-blinded trial, 62 adolescents with obesity aged 10–16 years old with insulin resistance received 2000 mg of metformin or placebo daily and physical training twice weekly over 18 months. Primary end points were change in body mass index (BMI) and insulin resistance measured by the Homeostasis Model Assessment for Insulin Resistance (HOMA-IR). Secondary end points were safety and tolerability of metformin. Other end points were body fat percentage and HbA1c.

**Results:**

Forty-two participants completed the 18-month study (66% girls, median age 13 (12–15) years, BMI 30.0 (28.3 to 35.0) kg m^−2^ and HOMA-IR 4.08 (2.40 to 5.88)). Median ΔBMI was +0.2 (−2.9 to 1.3) kg m^−2^ (metformin) versus +1.2 (−0.3 to 2.4) kg m^−2^ (placebo) (*P*=0.015). No significant difference was observed for HOMA-IR. No serious adverse events were reported. Median change in fat percentage was −3.1 (−4.8 to 0.3) versus −0.8 (−3.2 to 1.6)% (*P*=0.150), in fat mass −0.2 (−5.2 to 2.1) versus +2.0 (1.2–6.4) kg (*P*=0.007), in fat-free mass +2.0 (−0.1 to 4.0) versus +4.5 (1.3 to 11.6) kg (*P*=0.047) and in ΔHbA1c +1.0 (−1.0 to 2.3) versus +3.0 (0.0 to 5.0) mmol mol^−1^ (*P*=0.020) (metformin versus placebo).

**Conclusions:**

Long-term treatment with metformin in adolescents with obesity and insulin resistance results in stabilization of BMI and improved body composition compared with placebo. Therefore, metformin may be useful as an additional therapy in combination with lifestyle intervention in adolescents with obesity and insulin resistance.

## Introduction

Childhood obesity is an important pediatric health issue, with rising prevalence rates in almost all European countries, the United States and Canada.^1^ While it was recently reported that the prevalence of overweight and obesity may be stabilizing, percentages are still high, that is, 37.8% and 36.6% for 11- to 15-year-old boys and girls, respectively.^2^

Obesity increases the risk of insulin resistance (IR). Children with obesity with IR have a higher risk of developing type 2 diabetes mellitus,^3^ metabolic syndrome^3, 4^ and cardiovascular diseases.^5, 6^ Reduction of body mass index (BMI) is known to reduce the risk of developing these diseases.^7, 8, 9^ However, IR might be a limiting factor in losing weight in children and adolescents with obesity. In a study by Chiavaroli *et al.* the efficacy of a 1-year weight loss intervention programme in children with and without IR was evaluated. Children without IR achieved a reduction in BMI-s.d. score (BMI-SDS) following a weight loss intervention programme, whereas children with IR did not.^10^ Therefore, for children with obesity and IR additional (pharmacotherapeutic) therapies to lose weight are often considered.

Metformin is an antidiabetic drug, which reduces peripheral insulin resistance, increases the peripheral glucose uptake and decreases gluconeogenesis of the liver.^11^ Although metformin is approved for the treatment of type 2 diabetes mellitus from the age of 10 years onwards, there is an increasing number of prescriptions for off-label indications such as obesity, polycystic ovarian syndrome and type 1 diabetes mellitus, with percentages reported between 8 and 20%.^12, 13, 14^

The evidence for the efficacy of metformin in the treatment of obesity and IR in children is however scarce. A systematic review and meta-analysis of five randomized trials with a trial duration of 6 months (*n*=320) showed a moderate reduction in BMI (−1.42 kg m^−2^ (95% confidence interval 0.83–2.02)) and in IR measured by the Homeostasis Model Assessment for Insulin Resistance (HOMA-IR; −2.01 (95% confidence interval 0.75–3.26)).^15^ Long-term data on the efficacy of metformin is limited to one trial, which studied the efficacy of metformin over 48 weeks, and reported a small reduction in BMI.^16^ No other studies with treatment duration more than 6 months were identified.

Therefore, the aim of this randomized, double-blinded, placebo-controlled trial in adolescents with obesity with IR is to study the effect of metformin versus placebo on the change in BMI and in IR (measured by HOMA-IR) after 18 months. Secondary objectives included safety and tolerability, as well as change in body fat percentage, HbA1c, quality of life and physical fitness after 18 months.

## Materials and Methods

A brief description of the methods is provided here, as the study protocol has been published elsewhere.^17^

### Study design and participants

**Figure 1:**
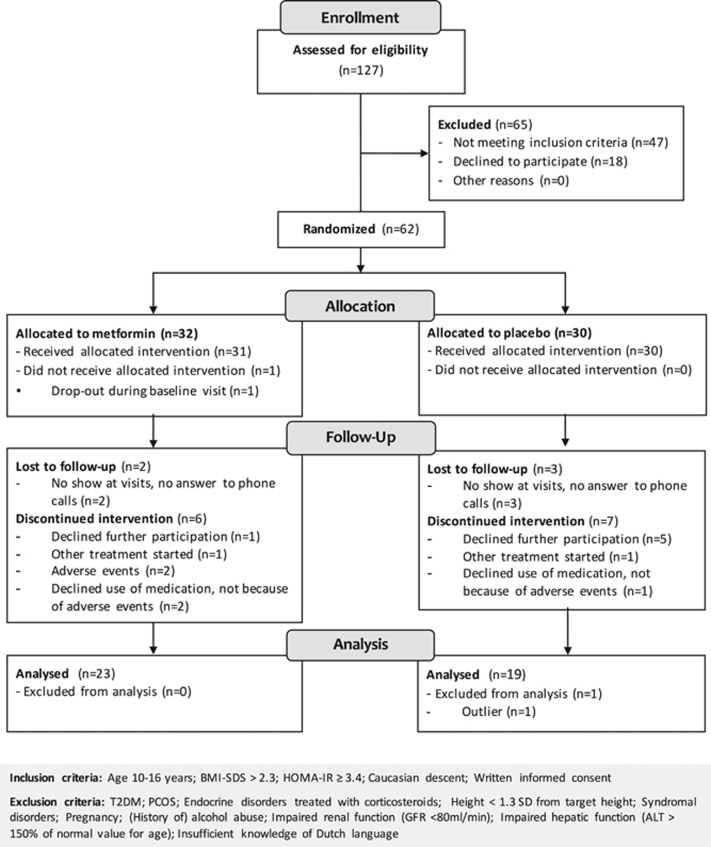
Consort flow diagram.

In this 18-month multicentre randomized, double-blinded, placebo-controlled trial (ClinicalTrials.gov number NCT01487993), participants were recruited at the pediatric outpatient clinics of the participating study centres (St Antonius Hospital in Nieuwegein/Utrecht (July 2011–March 2014) and Jeroen Bosch Hospital, ‘s Hertogenbosch (November 2012–March 2014), The Netherlands). For inclusion and exclusion criteria see Figure 1. All clinical measurements were performed in the pediatric outpatient clinics or day-care wards of these hospitals; the fitness tests were performed at the physical therapy outpatient clinic of the St Antonius Hospital and at the Sports Medical Centre of the Jeroen Bosch Hospital. The study protocol was approved by the Medical Ethical Committee of the St Antonius Hospital, Nieuwegein/Utrecht, the Netherlands, and written informed consent was obtained from the parents and if applicable, from the children (aged ⩾12 years). From the younger children, oral consent was obtained. All procedures were in accordance with the Declaration of Helsinki and the Medical Research Involving Human Subjects Act (WMO) of the Netherlands.

### Randomization and blinding

Consecutive study numbers for eligible participants corresponding with the randomization code and medication number (for example, study number 1, corresponds with randomization number 1 and medication number 1) were allocated. The randomization schedule (in blocks of 20 per study centre) was generated by the department of Clinical Pharmacy of the St Antonius Hospital, using PASW Statistics 18.0 (SPSS Inc., Chicago, IL, USA). Both subjects and study staff were blinded during the 18-month treatment period. The randomization code was kept secured in the department of Clinical Pharmacy. The blind was not broken for any of the participants.

### Sample size

For ΔBMI, a group sample size of 47 per group was calculated to have 90% power with a significance level of 5% to detect a difference of 2.94% in BMI (ΔBMI ±1 kg m^−2^). For ΔHOMA-IR, a group sample size of 60 participants per group was found to detect a difference of 1.6 in HOMA-IR, with a significance level of 5 and 90% power. Taking a dropout of 20% into account, the sample size was set at 144 participants.

### Interventions

#### Medication

All participants received either immediate-release metformin 500 mg tablets (Centrapharm, Etten-Leur, The Netherlands) or identical placebo tablets (Apotheek Haagse Ziekenhuizen, Den Haag, The Netherlands) in an increasing dosing regimen, with a maximum dose of two tablets twice daily in the fourth study week. Subjects were advised to take the medication during or after breakfast and dinner. In case of gastro-intestinal complaints, the dosage was reduced to the last well-tolerated dose. After symptoms had disappeared, the dosage was again increased to the maximum of two tablets twice daily, if tolerated. To estimate medication compliance, pill counts were performed on returned medication packages during each hospital visit (every 3 months).

#### Physical training

Physical training by a physical therapist was offered twice weekly to all participants. During the monthly phone calls and three monthly visits participants were encouraged to attend these trainings.

### Outcomes

#### Primary outcome measures

Primary end point was the change in BMI after 18 months (ΔBMI). BMI was calculated as weight (kg)/((height (m))^2^, and was assessed every 3 months. The corresponding age- and sex-adjusted BMI, the BMI-SDS, was calculated by the ‘TNO Groeicalculator voor professionals' (https://groeiweb.pgdata.nl/calculator.asp). As second primary outcome, ΔHOMA-IR over 18 months (HOMA-IR=fasting plasma glucose(mmol l^−1^) × fasting plasma insulin(mU l^−1^)/22.5) was evaluated.^18^ Fasting plasma glucose and fasting plasma insulin were measured and HOMA-IR was calculated every 3 months.

#### Secondary outcome measures

Secondary end points were safety and tolerability of metformin. Safety outcome measures were renal and hepatic function tests, measured at baseline and every 3 months during treatment. Vitamin B12 levels were measured, and levels <140 pmol l^−1^ were defined as abnormal. Tolerability was assessed by the amount of observed adverse events, and by the achieved maximum dosage levels. The reasons for dropout of participants were registered.

#### Other outcome measures

Other end points were change in body fat percentage measured by bio-impedance analysis using a leg-to-leg bio-impedance analysis measurement, and HbA1c after 18 months. Furthermore, change in quality of life assessed using a validated Dutch translation of the Impact of Weight on Quality of Life-Kids (IWQOL-kids) questionnaire,^19, 20^ and change in physical fitness assessed during validated fitness tests after 18 months were analysed. Participants were asked to fulfil a dietary diary at baseline, 9 months and 18 months to calculate caloric intake.

### Statistical analysis

All participants who started treatment (that is, they used at least one tablet of metformin or placebo) and finished follow-up of 18 months were analysed. As most parameters were not normally distributed, data are reported as medians with interquartile ranges. To assess the effect of metformin versus placebo after 18 months of treatment on the continuous scales, the Mann–Whitney *U*-test was applied. To compare the frequencies of categorical, dichotomous data, a *χ*^2^-test was used. All analyses have been conducted with SPSS for Windows version 22.0 (IBM Corp., Armonk, NY, USA).

## Results

### Participants

In all, 127 participants were assessed for eligibility (Figure 1). A total of 62 participants were allocated to metformin (*n*=32) or placebo (*n*=30), of which 42 could be included in the final analysis (Figure 1). One patient in the placebo group was excluded from the analysis, because this patient was an outlier with a change in BMI-SDS of −4.47. There was no difference in baseline age, sex, BMI, HbA1c and HOMA-IR between the participants lost to follow-up and participants who completed the 18-month treatment period (Supplementary Tables 1 and 2).

### Baseline characteristics

Baseline characteristics of the analysed participants are presented in Table 1. Overall, more girls than boys were included. In both groups, most participants were early pubertal and family history positive for obesity and diabetes mellitus was frequently reported. Median BMI at baseline was 29.8 (28.1–34.5) kg m^−2^ for the metformin and 30.5 (28.7–38.6) kg m^−2^ for the placebo groups, corresponding with an age- and sex-specific BMI-SDS of 3.10 (2.72–3.52) and 3.38 (3.10–4.20), respectively (Table 1).

### Medication compliance

Two participants did not return any medication packages during the study. In the metformin group 74% (17/22 participants) returned their medication boxes at least 4 times, versus 69% in the placebo group (13/18 participants). The returned boxes contained on average 28% and 24% of its content for the metformin and placebo groups, respectively. In case of full compliance, the remaining content should be 7% because all medication boxes had a surplus when dispensed.

### Effect on BMI and HOMA-IR

**Figure 2:**
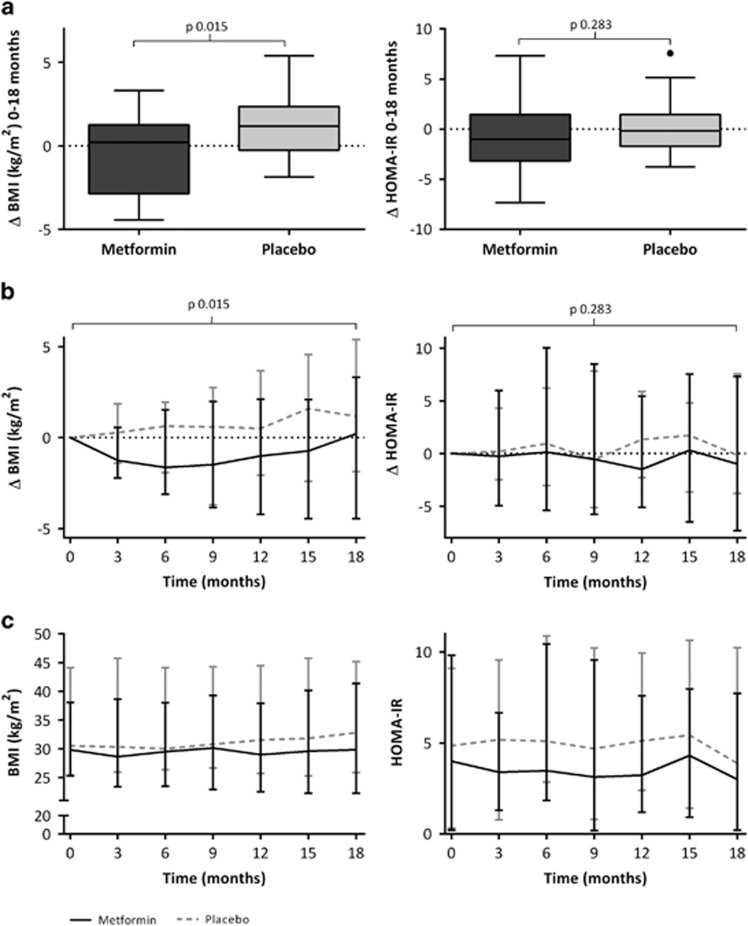
Effect of metformin on primary end points BMI and HOMA-IR after 18 months. (**a**) Change in BMI and HOMA-IR between *t*=0 and 18 months; (**b**) median ΔBMI and ΔHOMA-IR over time; (**c**) median BMI and HOMA-IR over time.

Table 2 presents the 18-month treatment results of metformin versus placebo; the absolute values for BMI and other parameters, as well as changes over 0–18 months are displayed. After 18 months, median ΔBMI was +0.2 (−2.9 to 1.3) kg m^−2^ in the metformin group versus +1.2 (−0.3 to 2.4) kg m^−2^ in the placebo group (*P*=0.015). Figure 2 shows that this difference between the two groups can be explained by a decrease in ΔBMI in the metformin group during the first 6–9 months of treatment and subsequent return to baseline values, which was not observed in the placebo group.

No significant difference was observed for ΔHOMA-IR after 18 months between both groups (Table 2). Figure 2 shows that in accordance with this lack of difference between the groups at 18 months, there is also no evidence for a difference in profile of ΔHOMA-IR over time during the study.

### Secondary end points safety and tolerability

#### Safety

No severe adverse advents occurred in either group. There were no derangements of renal or hepatic function (Table 3). In three participants of the metformin group, vitamin B12 levels below the threshold of 140 pmol l^−1^ were measured at 18 months (136, 117 and 108 pmol l^−1^, respectively).

#### Tolerability

Two out of nine participants lost to follow-up in the metformin group discontinued treatment because of adverse events. One patient had severe nausea despite dosage reductions. The other patient suffering from abdominal pain and discomfort, was not willing to try dosage reductions and terminated study participation. Four participants in the metformin group did not tolerate the maximum dose of 2000 mg daily because of adverse events; these participants used 1000 (*n*=3) or 1500 mg daily (*n*=1). In the placebo group, no participants dropped out because of adverse events.

Well-known side effects of metformin, nausea and diarrhoea, were reported in both groups during the study, but participants using metformin suffered significantly more from nausea (73.9%, *n*=17) than the participants receiving placebo (42.1%, *n*=8; *P*=0.037). Diarrhoea occurred in 60.9% (*n*=14) of the metformin users and 47.4% (*n*=9) of the placebo users (*P*=0.38; Table 3).

### Effect of metformin on HbA1c and body composition

**Figure 3:**
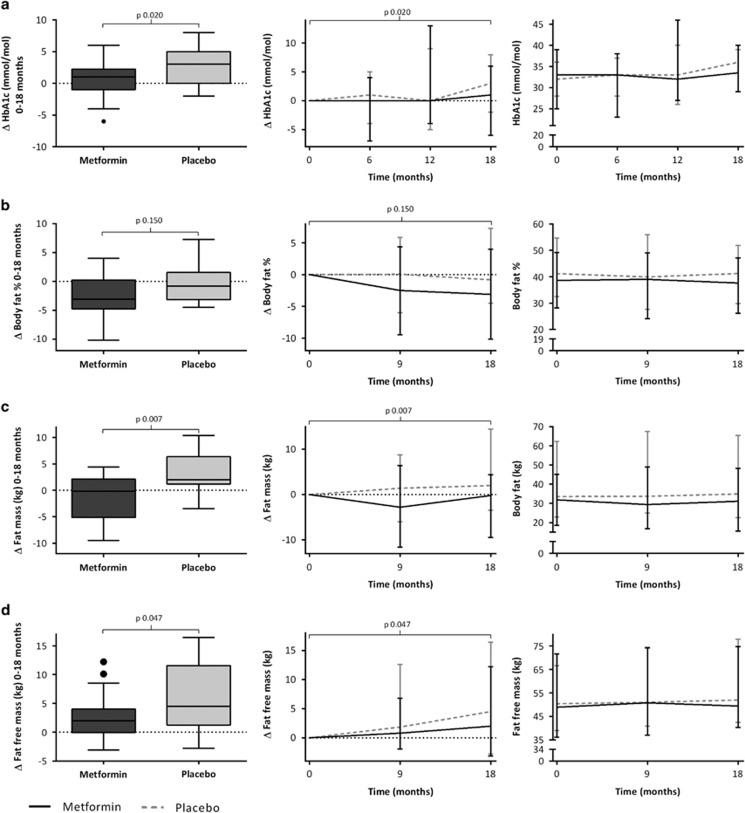
Effect of metformin on HbA1c and body composition after 18 months. Boxplots represent the Δ values between *t*=0 and 18 months. Graphs represent median values.

Table 2 and Figure 3 show that HbA1c increased in both groups, with a significantly larger increase in the placebo group (*P*=0.02). None of the participants had HbA1c values above the normal threshold after 18 months.

In the metformin group, fat mass decreased versus an increase in the placebo group (*P*=0.007) (Table 2; Figure 3). Concerning fat-free mass, in the metformin group the increase was+2.0 (−0.1 to 4.0) versus+4.5 (1.3 to 11.6) kg in the placebo group (*P*=0.047). There was no significant change in body fat percentage (Figure 3).

### Effect of metformin on quality of life and physical fitness

Table 2 shows results for quality of life measured by IWQOL-kids. For all sections and the total score, there was no difference in quality of life. Owing to a poor attendance at the physical tests after 18 months, physical fitness tests could only be analysed in a small subgroup (metformin *n*=15, placebo *n*=7; Table 2). At baseline, more than 50% of the participants completed the shuttle walk test, therefore the median score of the shuttle walk test was similar to the maximum score, and no significant differences were observed in this small subgroup. Dietary diaries were not completed and returned adequately, and therefore the caloric intake could not be calculated and analysed.

## Discussion

In this randomized, double-blinded, placebo-controlled trial in adolescents with obesity and insulin resistance, we found that assignment to the metformin group was associated with an initial decrease in BMI over the first 6–9 months of treatment after which BMI returned to baseline level, whereas BMI increased in placebo users. Changes in body composition and HbA1c over 18 months were also in favour of metformin. In contrast, in the placebo group, a steady increase in BMI was observed over 18 months. No serious adverse events were reported and most participants tolerated metformin up to 1000 mg twice daily; only two participants discontinued treatment because of adverse events.

Our study is the first study in an obese non-diabetic pediatric population reporting on the long-term effect (>1 year of treatment) of metformin on BMI. Beneficial effects on BMI upon short-term treatment with metformin have been reported before by Burgert *et al.*,^21^ who reported upon 4-month treatment a reduction in BMI of −0.9 kg m^−2^ (95% confidence interval −2.0 to 0.3 kg m^−2^) versus an increase in BMI of +1.2 kg m^−2^ (−0.1 to 2.4 kg m^−2^) in placebo. Upon 48 weeks of treatment, Wilson *et al.*^16^ reported a significant reduction in BMI of −0.9±0.5 kg m^−2^ for the metformin group versus +0.2±0.5 kg m^−2^ in the placebo group (*P*=0.03). Comparing our results with these previous short-term results, it seems that our results after 12 months closely resemble them (that is median ΔBMI −1.0 (−3.4 to 0.6) kg m^−2^ for metformin versus +0.6 (−0.2 to 2.1) kg m^−2^ for placebo, Figure 2)). In our study, where we report on treatment effects after 18 months, the difference between metformin and placebo remained significant even though it seems that BMI values return to baseline in the metformin group. However, in the placebo group there was no evidence of a decrease in BMI (Figure 2). An intriguing question is therefore how BMI will change over time after these 18 months. Lavine *et al.* treated children with obesity and non-alcoholic fatty liver disease for 96 weeks with metformin.^22^ However, the treatment in this study was not primarily focused on weight loss, and participants did not receive lifestyle intervention. They reported changes in BMI after 96 weeks of +1.3 (0.6–2.0) kg m^−2^ for metformin versus +1.9 (1.1–2.7) kg m^−2^ for placebo (*P*=0.25).^22^ This finding illustrates that metformin without lifestyle intervention may not be effective in changing BMI. As a consequence, follow-up results of our study (open-label results) upon 36 months' treatment with metformin with lifestyle intervention will need to be awaited.^17^ Until then, in our opinion, lifestyle intervention remains an important part of obesity treatment to which metformin therapy over 18 months seems to be of added value to reduce BMI.

In our study, two participants discontinued treatment and four participants received a reduced dosage because of adverse events, even though there were no serious adverse events or derangements in hepatic and renal function tests. These findings are comparable to the study of Wilson *et al.*^16^ of 48 weeks, where one patient dropped out because of nausea. In studies where metformin was administered over 2–6 months, no severe adverse events, elevated hepatic or renal function tests, or decreased vitamin B12 were reported. Concerning vitamin B12, in our study, three participants in the metformin group had decreased vitamin B12 levels and therefore monitoring of vitamin B12 levels upon long-term use of metformin should be considered. In all studies nausea and diarrhoea were the most frequently reported side effects.^21, 23, 24, 25, 26, 27, 28, 29^ Even though these side effects of metformin are mostly mild and self-limiting, a small number of participants (6%) did not tolerate metformin because of these side effects.^28^ It is known that the incidence of gastro-intestinal side effects is higher in patients using immediate-release metformin compared with extended-release metformin.^30, 31, 32^ Therefore, the use of extended-release metformin could be considered in the small number of patients with serious gastro-intestinal side effects. From this study, it seems that safety and tolerability of long-term metformin treatment is comparable to short-term treatment (6 months to 48 weeks), with no serious adverse events and only a small percentage of participants who do not tolerate metformin.

In the current study, participants treated with metformin were found to have an improved body composition measured by bio-impedance analysis after 18 months, with a decrease in fat mass and increase in fat-free mass compared with placebo. In the placebo group, the change in fat-free mass was larger than the change in fat mass. The placebo group has a larger increase in height (Table 2) during the 18 months; the increase in fat-free mass might be related to this increase in height. We assume that this increase in height, and therewith in fat-free mass, is caused by a difference in pubertal stage during the study. At *t*=18months, in the metformin group 38.1% was pubertal (Tanner stage 2–4) and 57.1% postpubertal (Tanner stage 5), compared with 64.7% pubertal and 35.3% postpubertal in the placebo group. In the metformin group an increase in fat-free mass in accordance with their increase in height over 18 months was found, without an increase in fat mass, resulting in a stable BMI. Also in other studies, a favourable effect of metformin on body composition (measured by dual-energy X-ray absorptiometry or bio-impedance analysis) after 2–11 months of treatment, compared with placebo was reported.^16, 21, 24, 29^ In adults, a decrease in body fat percentage was related to a decrease in systolic and diastolic blood pressure and cholesterol levels.^33^ Therefore, a change in body composition during metformin treatment might have a positive influence on cardiovascular risk factors.

A limitation of our study is the number of included participants. For the primary end point (ΔBMI), 66% of the targeted number of participants was included, while for the change in HOMA-IR this percentage was only 43%, despite a prolongation of the inclusion period by 1.5 years. Furthermore, the dropout rate was 32%, whereas a dropout rate of 20% was anticipated. This high dropout rate illustrates the difficulties in motivating adolescents with obesity for long-term treatment and follow-up. This difficulty is underlined by the poor attendance at physical fitness tests and by the dietary diaries, which had limited completeness and reliability. Frequent phone calls and written reminders by the study staff did not improve the compliance. The low number of included participants and high dropout rate could have resulted in insufficient power to statistically test our hypotheses. However, although our study has less power than anticipated, we were able to detect a significant effect of metformin on the primary outcome measure (ΔBMI). For comparison, with respect to the IR outcome, other studies with sufficient power did not find an effect on IR after 6 months and 48 weeks either.^16, 25, 28^ Another limitation is the measurement of IR. All participants had HOMA-IR ⩾3.4 during the screening for eligibility. At baseline, which was planned within a few weeks from screening, some participants had HOMA-IR values <3.4, while still being obese (BMI-SDS >2.3). As a possible explanation for this finding, participants may not tell the truth about their fasting state during the screening. Another reason may be the large coefficient of variation that has been reported for fasting insulin.^34, 35^ As HOMA-IR is based on fasting insulin, HOMA-IR will vary as well resulting in HOMA-IR<3.4, thereby explaining the lack of difference in this parameter.

## Conclusion

In conclusion, long-term treatment with metformin in adolescents with obesity and insulin resistance results in a stabilization of BMI and improved body composition compared with placebo. Therefore, metformin may be considered a safe additional therapy in combination with lifestyle intervention.

## Figures and Tables

**Table 1:** Baseline characteristics of the analysed participants

	*Metformin (*n=*23)*	*Placebo (*n=*19)*
*Clinical measurements*		
Age (years)	13.6 (12.6 to 15.3)	12.0 (11.3 to 14.0)
		
*Gender,* n *(%)*		
Boys	6 (26.1)	8 (42.1)
Girls	17 (73.9)	11 (57.9)
Height (cm)	162.9 (159.0 to 168.0)	162.0 (160.0 to 166.0)
Height-SDS	−0.08 (−0.65 to 0.71)	0.51 (−0.15 to 1.34)
Weight (kg)	82.2 (75.4 to 92.7)	86.1 (74.0 to 103.0)
BMI (kg m^−2^)	29.8 (28.1 to 34.5)	30.5 (28.7 to 38.6)
BMI-SDS	3.10 (2.72 to 3.52)	3.38 (3.10 to 4.20)
Hip circumference (cm)	101.0 (93.0 to 107.8)	100.8 (96.9 to 112.3)^a^
Waist circumference (cm)	97.0 (94.0 to 106.0)	103.8 (100.0 to 119.4)^a^
Waist-to-hip ratio	1.00 (0.95 to 1.04)	1.05 (0.96 to 1.10)^a^
Systolic blood pressure (mm Hg)	118 (115 to 124)	119 (113 to 126)
Diastolic blood pressure (mm Hg)	69 (61 to 72)	67 (57 to 77)
		
*Tanner stage,* n *(%)*		
Prepubertal (Tanner stage 1)	3 (13.0)	3 (16.7)
Pubertal (Tanner stage 2–4)	17 (74.0)	12 (66.6)
Postpubertal (Tanner stage 5)	3 (13.0)	3 (16.7)
		
*Family history, first and/or second degree,* n *(%)*		
Obesity	20 (86.9)	16 (84.2)
Diabetes mellitus	15 (65.2)	9 (47.4)
Hypercholesterolemia	14 (60.9)	9 (47.4)
Hypertension	16 (69.5)	13 (68.4)
Cardiovascular disease	14 (60.9)	14 (73.7)
		
*Highest level of education,* n *(%)*		
Participant		
Lowest	4 (17.4)	8 (42.1)
Low	16 (69.6)	7 (36.8)
Middle	3 (13.0)	4 (21.1)
High	0 (0)	0 (0)
Father		
Lowest	2 (8.7)	1 (5.3)
Low	9 (39.1)	5 (26.3)
Middle	7 (30.4)	9 (47.4)
High	3 (13.0)	2 (10.5)
Unknown	2 (8.7)	2 (10.5)
Mother		
Lowest	0 (0)	3 (15.8)
Low	10 (43.5)	10 (52.6)
Middle	8 (34.8)	4 (21.1)
High	4 (17.4)	0 (0)
Unknown	1 (4.3)	2 (10.5)
		
*Biochemical measurements*		
Glucose 0′ (mmol l^−1^)	4.8 (4.7 to 5.0)	4.8 (4.5 to 5.0)
Glucose 120′ (mmol l^−1^)	6.0 (5.6 to 6.6)	5.9 (4.8 to 7.2)
Insulin 0′ (mU l)	18.0 (11.0 to 27.0)	23.0 (12.0 to 26.0)
Insulin 120′ (mU l^−1^)	94.0 (63.0 to 138.0)	90.0 (54.0 to 128.0)
HOMA-IR	4.00 (2.30 to 6.36)	4.85 (2.40 to 5.78)
HbA1c (mmol mol^−1^)	33 (31 to 34)	32 (31 to 34)
Cholesterol (mmol l^−1^)	4.8 (3.9 to 5.3)	4.4 (4.1 to 5.0)
HDL (mmol l^−1^)	1.10 (1.02 to 1.22)	1.16 (1.03 to 1.36)
LDL (mmol l^−1^)	2.9 (2.3 to 3.3)	2.4 (2.1 to 3.2)
TG (mmol l^−1^)	1.4 (0.9 to 1.6)	1.4 (1.0 to 1.8)
Total cholesterol/HDL ratio	4.3 (3.4 to 4.7)	3.8 (2.7 to 5.0)
ALT (U l^−1^)	19 (15 to 26)	21.5 (14.5 to 28.5)
Kreatinin (μmol l^−1^)	51 (48 to 55)	50 (47 to 56)
Vitamin B12 (pmol l^−1^)	365 (267 to 420)	336 (280 to 492)
		
*Bio-impedance*		
Body fat (%)	38.6 (36.5 to 43.2)	41.2 (36.9 to 44.1)^a^
Fat mass (kg)	31.8 (25.0 to 39.4)	33.6 (27.6 to 48.5)^a^
Fat-free mass (kg)	48.9 (45.4 to 53.0)	50.4 (42.6 to 54.5)^a^
		
*Quality of life by IWQOL-kids*	n=*22*	n=*15*
Section 1, physical comfort	83.3 (72.5 to 94.2)	73.3 (63.3 to 86.7)
Section 2, body esteem	75.6 (63.3 to 88.3)	68.9 (55.6 to 84.4)
Section 3, social life	86.7 (76.7 to 93.3)	86.7 (80.0 to 90.0)
Section 4, family relations	100 (95.8 to 100)	100 (90.0 to 100)
		
*Physical fitness*	n=*18*	n=*13*
Shuttle walk test, distance in m	1500 (1138 to 1500)	1500 (955 to 1500)
9 m sprint test (s)	2.47 (2.35 to 2.60)	2.70 (2.05 to 3.00)
10 × 5 m sprint test (s)	20.63 (19.21 to 23.45)	20.28 (19.15 to 22.10)
Situps in 30 s (*n*)	21 (17 to 30)	17 (14 to 19)
Time to stand up from supine position	2.10 (1.80 to 2.84)	2.40 (1.90 to 2.96)

Abbreviations: ALT, alanine aminotransferase; BMI, body mass index; HDL, high-density lipoprotein; HOMA-IR, homeostasis model assessment for insulin resistance; IWQOL, impact of weight on quality of life; LDL, low-density lipoprotein; SDS, s.d. score; TG, triglyceride.

Reported values are median (interquartile range) or numbers (%).

a *n*=18.

**Table 2:** Treatment effects of metformin versus placebo after 18 months

	*Metformin (*n=*23)*		*Δ*T=*18*–T=*0*	*Placebo (*n=*19)*		*Δ*T=*18*–T=*0*	*Metformin versus placebo,* P*-value*
	T=*0*	T=*18*		T=*0*	T=*18*		*Δ*T=*18*–T=*0*
*Primary outcomes*							
BMI	29.8 (28.1 to 34.5)	29.9 (26.3 to 33.6)	0.2 (−2.9 to 1.3)	30.5 (28.7 to 38.6)	32.8 (29.3 to 40.4)	1.2 (−0.3 to 2.4)	**0.02**
BMI-SDS	3.10 (2.72 to 3.52)	2.90 (2.34 to 3.39)	−0.12 (−0.50 to 0.08)	3.38 (3.10 to 4.20)	3.29 (3.02 to 4.18)	0.04 (−0.24 to 0.10)	0.08
HOMA-IR	4.00 (2.30 to 6.36)	3.00 (2.00 to 4.29)	−1.00 (−3.17 to 2.25)	4.85 (2.40 to 5.78)	3.88 (2.86 to 5.56)	−0.16 (−1.71 to 1.48)	0.28
							
*Other outcomes*							
HbA1c	33.0 (31.0 to 34.0)	33.5 (30.8 to 34.3)	1.0 (−1.0 to 2.3)	32.0 (31.0 to 34.0)	36.0 (33.0 to 37.0)	3.0 (0.0 to 5.0)	**0.02**
Height (cm)	162.9 (159.0 to 168.0)	166.5 (160.3 to 171.0)	2.1 (0.5 to 6.6)	162.0 (160.0 to 166.0)	168.3 (163.7 to 171.3)	6.2 (2.5 to 8.7)	
Weight (kg)	82.2 (75.4 to 92.7)	83.4 (76.6 to 94.2)	1.6 (−4.2 to 5.9)	86.1 (74.0 to 103)	96.7 (79.0 to 111.0)	12.0 (2.7 to 17.0)	
Bio-impedance		*n=21*			*n=17*		
Body fat %	38.6 (36.5 to 43.2)	37.6 (30.9 to 40.9)	−3.1 (−4.8 to 0.3)	41.2 (36.9 to 44.1)	41.2 (37.7 to 46.9)	−0.8 (−3.2 to 1.6)	0.15
Fat mass (kg)	31.8 (25.0 to 39.4)	31.1 (22.6 to 37.6)	−0.2 (−5.2 to 2.1)	33.6 (27.6 to 48.5)	34.9 (29.5 to 53.1)	2.0 (1.2 to 6.4)	**0.007**
Fat-free mass (kg)	48.9 (45.4 to 53.0)	49.4 (46.7 to 55.4)	2.0 (−0.1 to 4.0)	50.4 (42.6 to 54.5)	52.0 (48.9 to 65.1)	4.5 (1.3 to 11.6)	0.05
IWQOL-kids		*n=17*			*n=12*		
Total score	83.0 (76.7 to 91.9)	90.0 (81.5 to 98.1)	2.6 (0.2 to 5.7)	80.0 (72.6 to 85.2)	84.8 (77.8 to 90.0)	5.2 (−2.2 to 9.6)	0.94
Section 1	83.3 (72.5 to 94.2)	91.7 (79.2 to 100)	6.7 (0 to 13.3)	73.3 (63.3 to 86.7)	90.0 (76.7 to 93.3)	6.7 (3.3 to 21.7)	0.41
Section 2	75.6 (63.3 to 88.3)	82.2 (66.1 to 94.4)	2.2 (−2.2 to 5.6)	68.9 (55.6 to 84.4)	73.3 (67.8 to 87.8)	5.6 (−3.9 to 10.6)	0.66
Section 3	86.7 (76.7 to 93.3)	95.0 (86.7 to 99.2)	3.3 (0 to 6.7)	86.7 (80.0 to 90.0)	90.0 (86.7 to 95.0)	0 (−6.7 to 6.7)	0.25
Section 4	100 (95.8 to 100)	100 (95.8 to 100)	0 (0 to 3.3)	100 (90.0 to 100)	100 (95.0 to 100)	0 (0 to 5.8)	0.86
Fitness test			*n*=15			*n*=7	
Shuttle walk test (m)	1500 (1163 to 1500)	1500 (1415 to 1500)	0 (0 to 120)	1180 (850 to 1500)	1360 (1170 to 1500)	270 (−100 to 320)	0.52
9 m sprint (s)	2.48 (2.40 to 2.65)	2.53 (2.38 to 2.71)	0.01 (−0.14 to 0.12)	2.70 (2.00 to 3.09)	2.60 (2.47 to 3.04)	0.08 (−0.37 to 0.58)	0.46
10 × 5 m sprint (s)	20.63 (19.21 to 23.44)	20.58 (19.14 to 21.46)	−0.40 (−2.30 to 0.60)	21.00 (20.00 to 25.53)	21.10 (20.53 to 24.03)	−0.10 (−0.60 to 0.53)	0.46
Situps in 30 s (*n*)	21 (17 to 30)	24 (19 to 30)	0 (−11 to 8)	17 (14 to 19)	23 (19 to 25)	5 (4 to 11)	0.33
Time to stand up from supine position (s)	2.10 (1.80 to 2.84)	2.31 (2.13 to 2.58)	−0.05 (−0.33 to 0.61)	2.40 (1.90 to 2.96)	2.88 (2.26 to 2.93)	−0.03 (−0.32 to 1.24)	0.49

Abbreviations: BMI, body mass index; HOMA-IR, homeostasis model assessment for insulin resistance; IWQOL, impact of weight on quality of life; SDS, s.d. score.

All values are median (interquartile range). Bold entries are used for *P*-values which were below the significance level of <0.05.

**Table 3:** Safety and tolerability of metformin versus placebo

	*Metformin (*n=*23)*	*Placebo (*n=*19)*	P*-value*
*Safety,* n *(%)*			
ALT >69 U l^−1^ (girls) or >78 U l^−1^ (boys)	0	0	NA
GFR <60 ml min^−1^	0	0	NA
Vitamin B12 <140 pmol l^−1^	3 (13.0)	0	NA
			
*Tolerability* *Adverse events,* n *(%)*			
Nausea	17 (73.9)	8 (42.1)	**0.04**
Diarrhoea	14 (60.9)	9 (47.4)	0.38

Abbreviations: ALT, alanine aminotransferase; GFR, glomerular filtration rate; NA, not applicable. Bold entry is used for *P*-value which were below the significance level of <0.05.
