# Supplementary material for: Long-term treatment with metformin in obese, insulin-resistant adolescents: results of a randomized double-blinded placebo-controlled trial
Source: Nutr Diabetes. 2016 Aug 29;6(8):e228–. doi: 10.1038/nutd.2016.37 (PMC5022149; doi:10.1038/nutd.2016.37)
Supplement: Supplementary Table 1 [file nutd201637x1.doc]

Supplemental table 1: Comparison of baseline data of participants lost to follow up and participants who completed the first part of the study.

|  | **Follow up complete (n=43)** | **Lost to follow up (n=18)** | **P-value** |
| --- | --- | --- | --- |
| Age | 13.4 (11.7-15.2) | 12.38 (11.4-15.3) | 0.61 |
| Gender, n (%)   - Boys - Girls | 14 (82.4)  29 (65.9) | 3 (17.6)  15 (34.1) | 0.21 |
| BMI | 30.0 (28.3-35.0) | 32.4 (28.9-35.2) | 0.45 |
| BMI-SDS | 3.25 (2.86-3.65) | 3.44 (2.99-3.63) | 0.49 |
| HbA1c | 33 (31-34) | 31 (30-35) | 0.17 |
| HOMA-IR | 4.08 (2.40-5.88) | 4.10 (3.23-5.77) | 0.55 |
