# Supplementary material for: Long-term treatment with metformin in obese, insulin-resistant adolescents: results of a randomized double-blinded placebo-controlled trial
Source: Nutr Diabetes. 2016 Aug 29;6(8):e228–. doi: 10.1038/nutd.2016.37 (PMC5022149; doi:10.1038/nutd.2016.37)
Supplement: Supplementary Table 2 [file nutd201637x2.doc]

**Supplemental table 2: Baseline characteristics of all randomized participants**

Reported values are median (Interquartile range) or numbers (%).

|  | **Metformin (n= 31)** | **Placebo (n=30)** |
| --- | --- | --- |
| *Clinical measurements* |  |  |
| Age (yr) | 13.6 (11.8-15.2) | 12.9 (11.4-15.2) |
| Gender, n (%)   - Boys - Girls | 7 (22.6)  24 (77.4) | 10 (33.3)  20 (66.7) |
| Height (cm) | 162.0 (156.0-168.0) | 162.6 (159.4-166.3) |
| Height-SDS | -0.01 (-0.56-0.64) | 0.34 (-0.27-1.17) |
| Weight (kg) | 80.2 (72.4-92.7) | 87.5 (74.2-98.7) |
| BMI (kg/m2) | 30.3 (28.2-34.5) | 32.0 (28.9-36.3) |
| BMI-SDS | 3.20 (2.72-3.55) | 3.44 (3.10-4.02) |
| Hip circumference (cm) | 102.5 (94.5-108.0) | 103.4 (96.0-113.3) |
| Waist circumference (cm) | 99.0 (94.0-107.8) | 103.0 (99.5-114.0) |
| Waist-to hip ratio | 1.00 (0.95-1.06) | 1.04 (0.93-1.10) |
| Systolic blood pressure (mmHg) | 118 (115-124) | 121 (116-127) |
| Diastolic blood pressure (mmHg) | 68 (61-72) | 67 (59-76) |
| Tanner stage, n (%)   - Prepubertal (TS 1) - Pubertal (TS 2-4) - Postpubertal (TS 5) | 5 (16.1)  23 (74.2)  3 (9.7) | 5 (16.7)  19 (65.5)  5 (16.7) |
| *Family-history, first and/or second degree, n (%)* | | |
| Obesity | 28 (90.3) | 24 (80.0) |
| Diabetes mellitus | 21 (67.7) | 15 (50.0) |
| Hypercholesterolemia | 20 (64.5) | 14 (46.7) |
| Hypertension | 22 (71.0) | 20 (66.7) |
| Cardiovascular disease | 19 (61.3) | 19 (63.3) |
| *Highest level of education, n (%)* |  |  |
| Participant   - Lowest - Low - Middle - High | 7 (22.6)  20 (64.5)  4 (12.9)  0 (0) | 11 (36.7)  10 (33.3)  9 (30.0)  0 (0) |
| Father   - Lowest - Low - Middle - High - Unknown | 3 (9.7)  11 (35.5)  10 (32.3)  4 (12.9)  3 (9.7) | 4 (13.3)  9 (30.0)  11 (36.7)  4 (13.3)  2 (6.7) |
| Mother   - Lowest - Low - Middle - High - Unknown | 1 (3.2)  14 (45.2)  9 (29.0)  6 (19.4)  1 (3.2) | 5 (16.7)  12 (40.0)  10 (33.3)  1 (3.3)  2 (6.7) |
| *Biochemical measurements* |  |  |
| Glucose 0’ (mmol/l) | 4.8 (4.6-5.0) | 4.8 (4.5-4.9) |
| Glucose 120’ (mmol/l) | 5.8 (6.1-7.0) | 5.9 (5.1-6.9) |
| Insulin 0’ (mU/l) | 20.0 (13.0-27.0) | 18.0 (12.0-26.0) |
| Insulin 120’ (mU/l) | 103.0 (67.0-146.0) | 75.5 (52.5-131.0) |
| HOMA-IR | 4.09 (2.60-6.27) | 4.04 (2.52-5.59) |
| HbA1c (mmol/mol) | 33 (30-34) | 32 (30-34) |
| Cholesterol (mmol/l) | 4.7 (3.9-5.2) | 4.5 (4.1-5.0) |
| HDL (mmol/l) | 1.14 (1.02-1.27) | 1.14 (1.01-1.42) |
| LDL (mmol/l) | 2.9 (2.3-3.1) | 2.4 (2.1-3.2) |
| TG (mmol/l) | 1.4 (1.0-1.7) | 1.5 (1.0-1.8) |
| Total cholesterol/HDL-ratio |  |  |
| ALT (U/l) | 20 (16-28) | 21 (15-27) |
| Kreatinin (µmol/l) | 50 (48-55) | 52 (47-59) |
| Vitamin B12 (pmol/l) | 365 (267-429) | 337 (253-427) |
| *Bio-impedance* |  | *n=29* |
| Body fat (%) | 39.3 (36.9-44.0) | 41.5 (36.8-46.1) |
| Fat mass (kg) | 32.1 (27.1-39.4) | 36.1 (28.1-46.8) |
| Fat free mass (kg) | 47.7 (43.5-52.5) | 51.0 (44.3-54.7) |
| *Quality of Life by IWQOL-Kids* | *n=30* | *n=24* |
| Section 1, Physical comfort | 25.5 (21.8-28.3) | 24.5 (19.0-27.0) |
| Section 2, Body esteem | 32.5 (26.0-38.3) | 30.0 (25.3-36.8) |
| Section 3, Social life | 26.5 (23.0-29.0) | 26.0 (24.3-27.8) |
| Section 4, Family relations | 30.0 (29.0-30.0) | 30.0 (28.0-30.0) |
| *Physical fitness* | *n=22* | *n=16* |
| Shuttle walk test, distance in m | 1500 (1118-1500) | 1500 (1065-1500) |
| 9 meter sprinttest (sec) | 2.50 (2.40-2.69) | 2.69 (2.03-2.91) |
| 10x5 m sprinttest (sec) | 21.52 (19.30-23.94) | 20.28 (19.20-23.00) |
| Situps in 30 seconds (n) | 21 (18-30) | 20 (17-24) |
| Time to stand up from supine position | 2.25 (1.85-2.86) | 2.40 (1.80-2.72) |

Reported values are median (Interquartile range) or numbers (%).

*Abbreviations: SDS – standard deviation score; BMI – body mass index; HOMA-IR - homeostasis model assessment for insulin resistance; HDL – high density lipoprotein; LDL – low density lipoprotein; ALT – alanine aminotransferase; IWQOL – impact of weight on quality of life.*
